# A Cross-Device and Cross-OS Benchmark of Modern Web Animation Systems

**DOI:** 10.3390/jimaging12010045

**Published:** 2026-01-15

**Authors:** Tajana Koren Ivančević, Trpimir Jeronim Ježić, Nikolina Stanić Loknar

**Affiliations:** University of Zagreb Faculty of Graphic Arts, HR-10000 Zagreb, Croatia; trpimir.jeronim.jezic@grf.unizg.hr (T.J.J.); nikolina.stanic.loknar@grf.unizg.hr (N.S.L.)

**Keywords:** web animations, user interface design, live rendering, benchmarking, operating systems, mobile, FPS

## Abstract

Although modern web technologies increasingly rely on high-performance rendering methods to support rich visual content across a range of devices and operating systems, the field remains significantly under-researched. The performance of animated visual elements is affected by numerous factors, including browsers, operating systems, GPU acceleration, scripting load, and device limitations. This study systematically evaluates animation performance across multiple platforms using a unified set of circle-based animations implemented with eight web-compatible technologies, including HTML, CSS, SVG, JavaScript, Canvas, and WebGL. Animations were evaluated under controlled feature combinations involving random motion, distance, colour variation, blending, and transformations, with object counts ranging from 10 to 10,000. Measurements were conducted on desktop operating systems (Windows, macOS, Linux) and mobile platforms (iOS, Android), using CPU utilisation, GPU memory usage, and frame rate (FPS) as key metrics. Results show that DOM-based approaches maintain stable performance at 100 animated objects but exhibit notable degradation by 500 objects. Canvas-based rendering extends usability to higher object counts, while WebGL demonstrates the most stable performance at large scales (5000–10,000 objects). These findings provide concrete guidance for selecting appropriate animation technologies based on scene complexity and target platform.

## 1. Introduction

Web-based imaging and animation technologies have become integral to digital communication, interactive media, visualisation systems, and user interface design. As modern applications increasingly require consistent performance across different devices, understanding the rendering characteristics of various web technologies is essential. SVG is a true vector graphics format that offers resolution independence through explicit geometric shape definitions. In contrast, HTML/CSS and the Canvas API produce rasterised output: HTML/CSS elements are processed through the browser’s layout, paint, and compositing pipeline, while the Canvas API renders directly to a pixel-based bitmap surface. Although both HTML/CSS and Canvas rely on geometric calculations (such as layout boxes, paths, transforms, or border-radius), their rendered output is rasterised. WebGL differs from these approaches by using a GPU-accelerated pipeline that operates on geometric primitives and programmable shaders before rasterising the final image to the framebuffer. While prior studies have examined subsets of web animation technologies, platforms, or execution models, systematic cross-platform benchmarks that evaluate multiple browser-native animation paths under identical workloads and feature combinations remain limited. It should be noted that the animations evaluated in this study are intentionally designed as a stress-test workload. The benchmark emphasises repaint, compositing, and blending operations to expose scaling behaviour, execution-path bottlenecks, and stability limits under adverse rendering conditions, rather than to represent average-case user interface animation patterns.

The objective of this study is to evaluate how different web-based animation approaches behave across platforms, operating systems, and device classes. Using a consistent animated motif—circles undergoing translation, colour and opacity modulation—we assess responsiveness, computational load, and rendering stability. While many production user interface animations deliberately use transform-only motion and opacity changes to minimise repaint cost, the chosen workload provides an upper-bound characterisation of performance behaviour when such optimisations cannot be assumed.

The contribution of this research is to provide a comparative cross-platform evaluation of web-based vector and pixel animation technologies, a methodology for performance testing across heterogeneous environments, and insights relevant for imaging, visualisation, and high-performance graphical applications distributed through the web.

### 1.1. Overview of Previous Research

#### 1.1.1. Fundamental Overview and Technological Challenges of Web Animation

Early and comprehensive research on web animation technologies highlighted key issues related to the transmission, compression, and display of animation data on the web [1]. It has been shown that there is still no universal, standardized format that enables reliable and consistent processing of animations across different environments. Furthermore, the performance of web animations depends largely on network conditions, data volume, and the implementation specifics of individual browsers, which present major scalability limitations. These observations were also confirmed in one of the first empirical comparisons of animation technologies Flash, SVG, HTML5 Canvas, and JavaScript across multiple browsers [2]. The study clearly showed that HTML5 Canvas and JavaScript offer significantly better performance than Flash, particularly in terms of display speed and loading time. Based on this, the authors concluded that these technologies, along with SVG, represent the most suitable and portable solutions for web animations. Given the additional security and performance shortcomings, as well as the dependence on external plug-ins, Flash is now considered an outdated technology that is no longer used in modern web systems.

#### 1.1.2. Cross-Platform Frameworks and Performance Variability

During the same period, a study was published comparing the performance of the cross-platform frameworks MoSync, Titanium, jQuery Mobile, and PhoneGap in applications that make intensive use of animations [3]. The results show significant differences in execution speed, resource consumption, and the complexity of animation implementation. Titanium proved to be the most balanced solution. Hybrid approaches, such as jQuery Mobile and PhoneGap, experience significant performance degradation on mobile devices. Subsequent research on animations in cross-platform mobile applications confirms that animations are a key component of modern user interfaces [4]. However, their performance varies considerably between frameworks and platforms. iOS can reach a very high CPU load in certain animation scenarios, even in native apps. This demonstrates that the native approach does not guarantee stable performance.

These conclusions are also supported by studies examining the broader context of cross-platform solutions development. Interest in cross-platform frameworks is continuously growing [5]. Differences in tool architecture and frequent changes in mobile operating systems necessitate ongoing research. Tools must function reliably in heterogeneous Android and iOS environments [6]. Performance depends on the trade-off between the level of abstraction, access to native APIs, and platform architecture. Additional layers of abstraction often introduce measurable performance overhead [7]. Empirical analysis confirms this overhead, especially in animation-intensive operations. In these cases, cross-platform applications often consume more processor and memory resources than native solutions [8]. A systematic review confirms the persistent fragmentation between platforms and highlights the differing performance profiles of individual frameworks [9]. The results of a comparative analysis of Flutter and React Native, based on measurements of CPU and memory consumption during GUI operations, confirm that the performance of animation and interaction elements varies depending on the framework and execution environment, with no clear winner. This further justifies the need for detailed empirical benchmarking in related web-based animation systems [10,11]. These results clearly show that platform heterogeneity remains a persistent challenge. This is also true for web-based animation systems. Therefore, a cross-device and cross-OS benchmark, such as the one implemented in this paper, is necessary.

#### 1.1.3. 2D and 3D Web Animation Techniques and Performance

Analysis of contemporary animation techniques shows that CSS animations, particularly when using a single variable expression, achieve the best performance compared to popular JavaScript libraries [12]. This is because CSS directly leverages rendering engine optimizations, whereas JavaScript solutions introduce additional overhead. Performance research comparing HTML/CSS, SVG, p5.js, and WebGL confirms that traditional web technologies can provide the most stable display, while WebGL, especially on older hardware, can cause significant processor load [13]. The technological limitations of this research suggest the need for evaluations across a wider range of devices and operating systems. A study comparing the performance of CSS3, WAAPI, and Lottie animations further demonstrates that performance can deteriorate significantly when animations process a larger number of elements, and that browsers such as Safari may be more stable under load [14]. For interactive animations, it has been emphasized that achieving good performance depends on the compatibility of visual content complexity, code structure, and browser capabilities [15]. The key factor is the choice of DOM, Canvas, or WebGL technology, which must meet the display requirements and device load.

#### 1.1.4. 3D Visualization, AR, and Advanced Display Methodologies

In the field of 3D visualization, WebGL and WebXR have been identified as fundamental technologies for modern web visualizations, though they remain limited by available computing resources, network speed, and data formats [16]. A framework for assessing the maturity of visualization tools has been proposed, noting that web browsers are becoming a key platform for interactive visual analytics.

A methodology for the so-called “webization” of 3D content is presented in a paper that combines 3D models, point-cloud data, and HTML DOM elements in a unified environment [17]. This enables the display of complex 3D scenes without the need for additional plug-ins. Accordingly, a web application for displaying 3D robots uses a combination of WebAssembly and Babylon.js to create realistic animations in the browser [18]. The system allows users to test the robot’s functionality in a simulated home environment, highlighting the importance of affordable and high-performance web solutions.

#### 1.1.5. WebAssembly Performance in Animations and Computational Tasks

A comparison of WebAssembly and JavaScript in interactive animations demonstrates a significant difference in execution speed, with WebAssembly achieving almost instantaneous processing, while JavaScript takes much longer for the same task [19]. These results confirm the technological advantage of WASM in performance-demanding web applications and highlight its potential for complex interactive systems.

WebAssembly in modern web browsers can achieve significant speed improvements in computationally demanding tasks, confirming its suitability for real-time applications [20]. Comparisons of different WASM runtimes have shown that performance strongly depends on the chosen execution environment, which is particularly important in edge and serverless scenarios [21].

Studies focused on embedded and IoT environments [22,23] indicate that WebAssembly can be highly efficient even on devices with limited resources, but also emphasise that memory consumption optimizations and architectural adaptation are crucial for stable performance. The dependence of performance on hardware architecture is further confirmed by research showing that the use of a specialised hardware accelerator can multiply the execution speed of WebAssembly code and significantly improve performance in resource-limited systems [24].

#### 1.1.6. Performance Perception and User Experience

Research on user perception shows that dynamic animations during page loading can positively influence perceived speed, as they divert attention from the passage of time and reduce the subjective feeling of waiting [25]. This indicates that animations affect not only technical performance but also the user’s experience of interaction.

#### 1.1.7. Synthesis and Relevance to Cross-Device Benchmarking

A review of these studies shows that the performance of web animations varies significantly depending on technology, browser, operating system, and hardware. This fragmentation clearly demonstrates the need for systematic testing across different environments. Therefore, cross-device and cross-OS benchmarking, as conducted in this paper, is essential for understanding the actual performance of modern web animation systems.

Although web animation is widely used in modern interfaces, data visualisation, and interactive educational content, it has received relatively little attention in systematic scientific studies. This is partly due to the interdisciplinary nature of the field, which spans web development, computer graphics, and system-level browser engineering, as well as the rapid evolution of rendering technologies and platforms. Consequently, existing research often focuses on isolated techniques or specific platforms, while comprehensive, cross-technology and cross-platform performance analyses remain rare. From a scientific perspective, web animation performance is not merely a design concern but a systems-level issue involving rendering pipelines, resource scheduling, and platform-dependent execution behaviour.

The novelty of this research lies in a systematic and comparative evaluation of web animation technologies across multiple rendering models and platforms under controlled and incrementally combined animation features. The primary motivation is to address the lack of reproducible, cross-platform performance benchmarks for web animation, despite its growing practical importance. By analysing CPU usage, GPU usage, and frame rate across increasing object counts, this study provides quantified, practice-oriented guidance for selecting appropriate animation technologies based on scene complexity and target platform, while also contributing methodological insight for future performance-oriented web animation research.

## 2. Methodology

For purposes of this research, we developed an application for parametrically generating web animations using native web technologies, of which we count eight applicable approaches:

- CSS animations of absolutely positioned DOM elements in the HTML structure;
- CSS animations of DOM elements in the HTML structure using its transformation engine;
- CSS animations of DOM elements in the SVG structure;
- CSS animations of DOM elements in the SVG structure using CSS’s transformation engine;
- JavaScript animations of absolutely positioned DOM elements in the HTML structure using its *requestAnimationFrame* loop engine;
- JavaScript animations of DOM elements in the SVG structure using its *requestAnimationFrame* loop engine;
- JavaScript-generated animations based on rendering images onscreen using the Canvas API and JavaScript’s *requestAnimationFrame* loop engine;
- WebGL rendered animations based on rendering images onscreen using the Canvas API and JavaScript’s *requestAnimationFrame* loop engine.

In this study, these approaches are compared at the level of browser rendering and execution paths rather than low-level API equivalence. The evaluated approaches represent distinct execution models within modern browsers, including compositor-driven CSS animations, JavaScript-driven DOM updates executed via *requestAnimationFrame* (RAF) calls, immediate-mode Canvas rendering, and GPU-accelerated WebGL rendering. Although different APIs and abstraction layers are used to implement these approaches, the comparison focuses on their observable performance under an identical perceptual workload.

**Figure 1:**
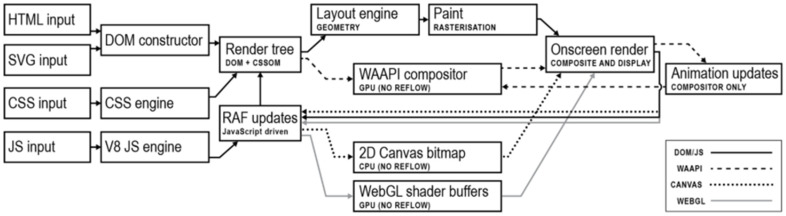
Execution flow of web animations depending on the choice of rendering technologies.

Each of the above-mentioned animation technologies is native to all modern web browsers, and each presents its unique approach to content display and animation rendition, as shown in Figure 1. All evaluated animation approaches rely on browser-native rendering paths supported by modern web browsers, although they differ in their execution models and abstraction levels.

All approaches require some basic DOM inputs and rely on default or user-modified CSS definitions, but their animation-rendering paths diverge early as they progress towards onscreen frame rendering according to the technology used to run animations. These divergencies result in performance differences during execution. HTML elements are objects present in the DOM and are subject to surrounding automated mechanisms for layout responsiveness. Absolutely positioned HTML elements are excluded from those mechanisms and require programmatic coordinate assignments to determine their position on screen. SVG elements are similar in that matter, as they are part of the DOM structure and require coordinates, but are rendered through a different SVG engine, which lacks certain optimisation techniques available in HTML’s pure-blocks layout engine. CSS features two distinct mechanisms for rendering animations. One is its transition mechanism that can interpolate smooth transitions between any two states for any numerically valued DOM attribute. It is the basis of the CSS animation API, and its execution is primarily CPU-based. Another is the CSS transformation mechanism, which does not act on browsers’ automatic layout mechanisms but renders on top of them, and is optimized through the browser’s compositing layer system and promoted to GPU rendering. Both of those mechanisms are applicable for animating HTML or SVG DOM elements. JavaScript is, in this scenario, a browser-run programming language that can calculate representations for the animation of any number of DOM attributes and then dynamically apply them to the DOM presentation. In this context, it is executed on the CPU in a single-threaded manner. Web Animation API (WAAPI) provides an interface for JavaScript inputs and is used in this application to generate keyframe animation definitions to be run on the CSS engine. The Web Animation API is used only as a mechanism for defining keyframe animations and does not constitute a separate experimental condition. Performance attribution in this study is based on the underlying execution path: animations executed by the CSS compositor are categorised as CSS-based, while animations requiring per-frame JavaScript execution are categorised as JavaScript-driven. The Canvas API offers a JavaScript-based, immediate-mode rendering interface that operates independently of the DOM, enabling direct manipulation of pixel data within a rasterised drawing surface. Unlike DOM-based methods, Canvas does not retain scene structure and requires explicit redrawing for each frame. Although the Canvas API is CPU-driven, it can act as a rendering target for WebGL contexts, which provide GPU-accelerated graphics through programmable shaders. In this study, WebGL rendering is accessed via the three.js library, which abstracts low-level shader management and GPU communication. As a result, measured WebGL performance includes the framework-level overhead introduced by the three.js abstraction layer and should be interpreted as representative of practical WebGL-based animation workflows, rather than minimal hand-written WebGL implementations.

An application developed for testing the performance of different web native animation technologies presents a simple user interface for defining parameters that determine the complexity of animation to be rendered on screen. The base concept of animation to be tested is a composition of an arbitrarily large number of simple circular elements of the same size and colour, and laid out in a grid-like manner across the screen. Each row accommodates 50 elements. Elements are animated in a four-segment animation, moving on a square-shaped path with a side length equal to the diameter of each circle. Additional parameters control the intensification of complexity starting from the base animation. The count parameter is of integer type and controls the number of elements to be accommodated in the render. The random motion parameter (M) is a Boolean that offsets each point of the square motion path by a random percentage of its size, ensuring that each circle gets its unique motion path, which prevents global optimizations. The parameter of distance (D) acts as a multiplier on those off-path divergences, ensuring the frequent overlap of various shapes. The parameter of random colour (C) is also a Boolean that adds to the animation requirement for reaching a different random colour on the end of each of the four segments of an animation loop. The parameter of blending does the same thing as that of random colour, but for elements’ opacity, making the rendering of elements’ overlaps resource-demanding. Transformations (T) is a Boolean parameter that lets users choose whether the HTML or SVG elements should be animated with CSS transitions on DOM attributes or CSS transformations.

For our research purposes, we defined several presets for generating animations to be used in taking measurements on different devices and operating systems. Those presets are labelled in shorthand for their parameter settings, as for example “M-D5-C-B-T” would mean animation with random movement and distance multiplier 5 enabled using CSS transformations, while also featuring random colour and blending keyframes interpolations. The evaluated feature combinations were constructed from commonly used animation components, including motion, spatial displacement, colour variation, blending, and geometric transformations. These features were incrementally combined to assess their individual and cumulative impact on performance.

The primary measurement grid comprises object counts of 10, 100, 500, 1000, 5000, and 10,000 elements, which are used consistently across all figures and quantitative analyses. Higher object counts (50,000, 100,000, and 1,000,000) were evaluated only as exploratory stress tests to illustrate scalability limits and are discussed qualitatively rather than included in the main benchmark results. Object counts were selected using a logarithmic progression with an arithmetic half-step to capture both typical real-world animation loads and high-complexity scenarios, enabling systematic analysis of performance scaling across different technologies.

To ensure the comparability of obtained test results, the generative application preloads and reads in all necessary scripts during its primary page load. In this way, any bandwidth or connectivity issues are made irrelevant for measuring its execution. Each possible animation is run by a single parent function, and all parent functions are developed to feature the same code snippets for creating loops and animation definitions per element and for executing these animations, varying only in direct calls to the respective animation API selected.

Application code and detailed documentation are made available for viewing, testing, or downloading on our public repository (see Supplementary section). Additional measures for obtaining reproduceable results were disabling of system caching; doing a hard reload before each next measurement (to kill off any remaining background processes); keeping the battery fully charged while testing on laptops and other mobile devices; monitoring CPU temperature and waiting for it to cool off if overheated; and taking the mean of obtained results for weighing them towards the objective metric of measured performance features.

Measurements were conducted on comparable high-performance business-class computers with three operating systems (Windows 11 25H2, macOS Tahoe, and Linux Pop!_OS 24.04 LTS), as well as on mobile devices: iPhone 17 Pro with iOS 26.1 and Samsung Galaxy A52s 5G with Android 14. Specifications for all devices and operating systems used are provided in the Supplementary section titled average_measured_data. The most significant device specifications are extracted and presented in Table 1. All devices and operating systems listed in Table 1 are officially released consumer hardware and publicly available operating system versions at the time of measurement.

For each OS, each web animation technology, variation of additional parameters, and for each iteration of circles, CPU, GPU, and FPS data were measured ten times (n = 10), and the resulting means are included in the final table (Supplementary section titled average_measured_data). All measurements were performed in Google Chrome. Browser versions are listed in Table 2.

**Figure 2:**
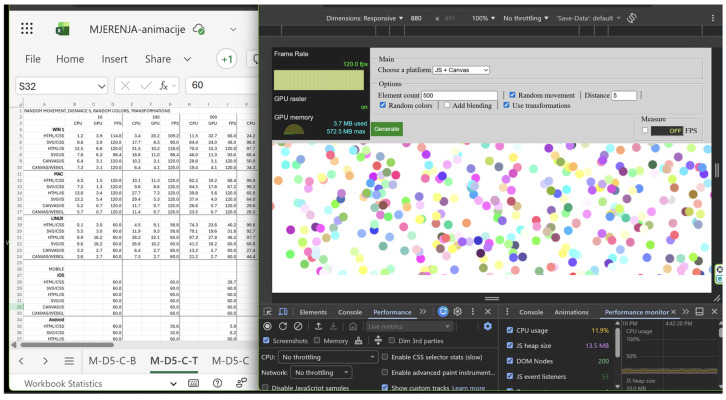
Working environment while performing measurements on Windows.

All data for desktop devices were collected using Chrome’s Developer tools (see Figure 2). Desktop platforms allowed for direct observation of CPU and GPU usage and were therefore analysed at the hardware utilisation level. Reported CPU values correspond to browser process-level utilisation observed during steady-state animation execution, while GPU values represent memory usage associated with the active rendering context. Measurements were sampled during animation runtime and aggregated as mean values for each run. Instrumentation was used solely for passive observation during data acquisition to minimise measurement interference. When measuring animation performance on mobile, we focused on measuring FPS across all combinations of animation technologies, number of elements, and additional animation functions included. On mobile, performance evaluation was limited to frame rate (FPS) measurements, as mobile browsers do not provide reliable CPU and GPU utilisation metrics to web applications. The script was developed to measure FPS directly from the execution of applications in the browser window. Accordingly, interpretations of thermal or scheduling behaviour are based on observable symptoms and should be considered speculative without direct system-level measurements. Figure 2 shows the workspace while performing measurements on Windows.

**Figure 3:**
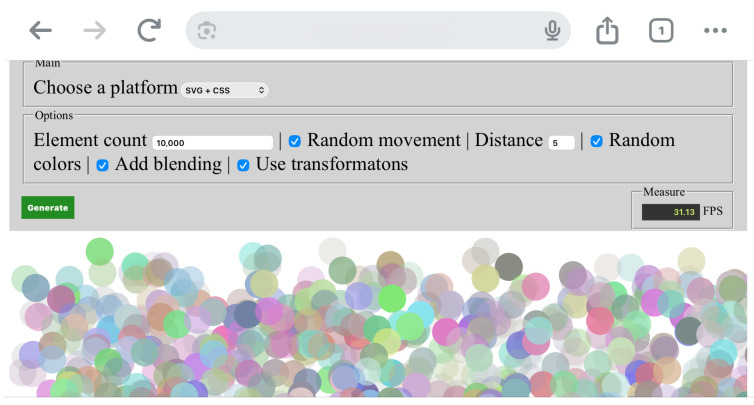
Working environment while performing measurements on iOS.

Figure 3 presents the working environment while performing measurements on iOS.

## 3. Results

The results in this research are divided into three major sections: CPU analysis, GPU analysis, and FPS analysis.

### 3.1. CPU Analytics

A central processing unit (CPU) can experience a heavy load with complex web animations where animated elements change position, as seen in the animations presented here. Although it might be expected that HTML/CSS and SVG/CSS would place the least load on the CPU, measured values across platforms and technologies show that, in all cases, JavaScript animations—HTML/JS and SVG/JS—produce the highest CPU load in this research. These technologies require continuous execution of JavaScript logic (*requestAnimationFrame*, attribute manipulation, position calculations, colour, and transformation), resulting in constant CPU usage. As the number of elements increases (1000, 5000, 10,000), CPU load rises exponentially and quickly reaches 100%, especially in combinations involving overlapping transparent elements. CSS animations (HTML/CSS and SVG/CSS) in this experimental environment show lower CPU usage than the JS approach, although CSS also involves significant reflow and repaint operations, and CPU usage remains unexpectedly high. The lower CPU load of CSS compared to JS is due to the browser’s ability to partially optimize style and layout calculations, whereas JS animations consume CPU resources for animation logic and additional invalidation of elements in each frame. Canvas/JS achieves lower CPU usage than the HTML/SVG JS approach, and in certain tasks even outperforms CSS animations, but still consumes significant CPU resources at higher iteration counts because the entire calculation of display attributes needed for rendering shapes on screen is driven by JavaScript before each frame execution. WebGL, by contrast, is arguably the most efficient technology in terms of CPU consumption. It offloads most of the processing to the GPU, which is why WebGL maintains the lowest CPU usage even with the largest numbers of animated elements featuring element-specific random movement paths, unique colour, and blending changes. Feature analysis shows that blending (B) increases CPU load the most across all technologies, especially JavaScript animations. The combinations C + B and C + B + T result in the highest CPU load, with JavaScript technologies being the most sensitive to increased complexity. The C component alone produces the highest CPU load with HTML/CSS and SVG/CSS, because colour changes in CSS are not GPU-optimised operations and trigger a repaint of all animated elements each time. As a large number of objects are animated in the test, each colour change causes thousands of repaint operations per frame, which quickly leads to high CPU usage.

**Figure 4:**
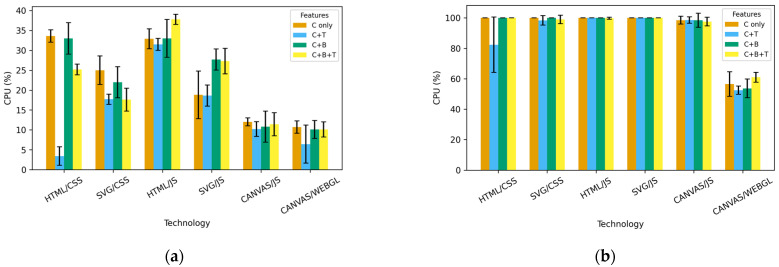
CPU across feature combinations and technologies on Windows OS: (**a**) 100 iterations and (**b**) 5000 iterations. Error bars indicate standard deviation across repeated runs (n = 10).

The research results on CPU behaviour while executing different web animations are shown in Figure 4. Figure 4a shows the CPU load for the technologies used for the animation and the included animation properties at 100 iterations, while Figure 4b shows the results at 5000 iterations. The analysis is comparative and benchmarking-oriented rather than inferential; therefore, the results are interpreted descriptively, and no formal hypothesis testing is conducted.

**Figure 5:**
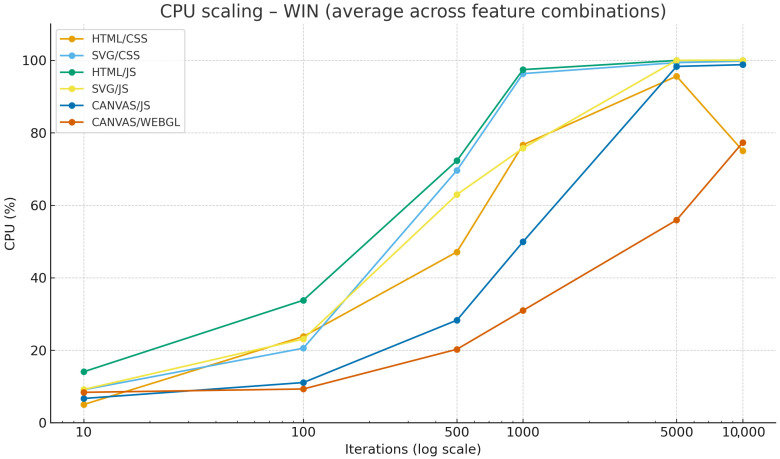
Comparison of CPU usage across web technologies and iterations on Windows OS (average across feature combinations).

Figure 5 shows the average CPU increase versus iteration increase for Windows OS. Among operating systems, Windows displays the most stable results and the least variability between iterations, while macOS and Linux exhibit slightly greater deviations.

### 3.2. GPU Analysis

From the measured values of GPU consumption across all platforms, technologies, and feature combinations (C, C + T, C + B, C + B + T), it is evident that DOM technologies—specifically HTML/CSS and SVG/CSS—occupy the most GPU memory. With a large number of simultaneously animated elements, the browser creates additional compositing surfaces and alpha-buffer layers, resulting in a continual increase in GPU memory usage. In all measurements, HTML/CSS produces the highest GPU values, closely followed by SVG/CSS, a pattern consistent across platforms. HTML/JS and SVG/JS, as expected for being CPU-reliant, exhibit moderate GPU load. Although the JS approach requires fewer internal layout layers than CSS, GPU memory still increases proportionally with the number of elements and the refresh rate. JavaScript animations generate fewer GPU layers than CSS variants but still maintain a relatively high GPU footprint, especially when blending is enabled.

**Figure 6:**
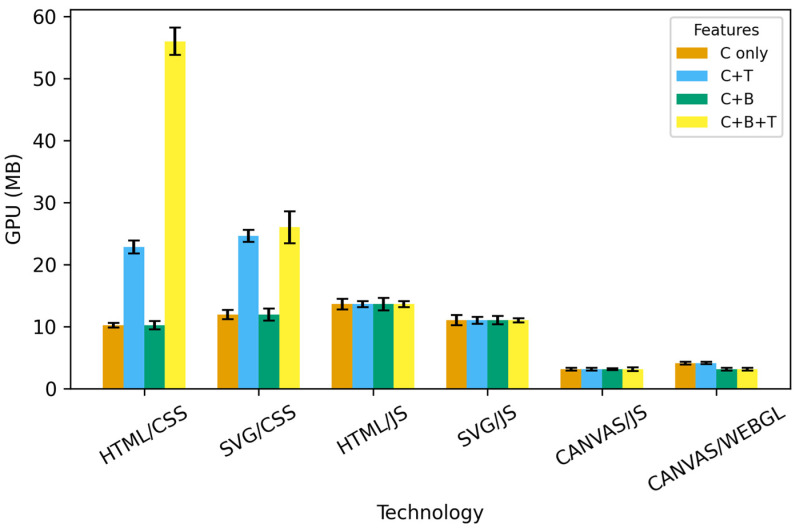
Comparison of GPU usage across web technologies and feature combinations on 5000 iterations. Error bars indicate standard deviation across repeated runs (n = 10).

A particularly high load is observed with the Linux operating system using HTML/JS at 10,000 repetitions. Canvas/JS and WebGL exhibit by far the lowest GPU consumption. With both technologies, all rendering occurs within a single graphics context (one canvas or one WebGL framebuffer), minimizing the number of textures and layers the GPU must manage. In all measurements, WebGL stands out as the technology with the lowest and most stable GPU usage, while Canvas uses slightly more GPU memory, but still significantly less than the HTML/SVG approach. An overview of GPU load across web technologies and feature combinations at 5000 iterations on Windows OS is shown in Figure 6.

Analysis of individual features shows that blending (B) increases GPU consumption the most, but primarily in DOM technologies (HTML/SVG, CSS, and JS variants). With Canvas and WebGL, the effect of blending is minimal because alpha operations occur within a single GPU context, without creating additional compositing surfaces. Transformations (T) have a relatively small impact on GPU consumption across all technologies.

**Figure 7:**
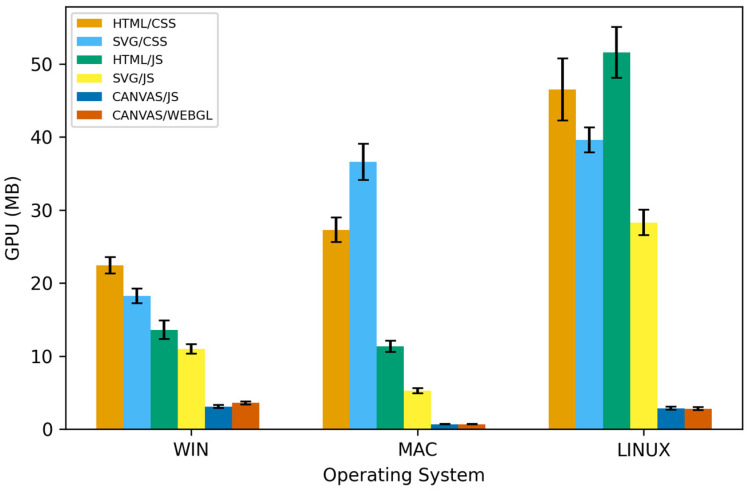
Comparison of GPU usage across web technologies and Desktop OS in the animation of 10,000 objects. Error bars indicate standard deviation across repeated runs (n = 10).

Regarding operating systems, Windows displays the most stable GPU results, while macOS and especially Linux exhibit slightly greater variations, likely due to differences in graphics drivers and the way the OS manages the GPU compositing pipeline. All trends remain consistent across different feature combinations. Figure 7 provides an overview of GPU load across animation technologies and operating systems at 10,000 iterations, with visible differences between operating systems.

### 3.3. FPS Analysis

The measured FPS values show that technologies and platforms behave very differently as the number of elements increases. WebGL consistently delivers the best performance in all conditions, maintaining the highest and most stable FPS values regardless of the number of repetitions or feature combinations. WebGL achieves a stable framerate even under maximum load, confirming its advantage in GPU-accelerated rendering. Canvas/JS also maintains stable FPS at low and medium loads, with the expected linear decrease as the number of elements increases. However, Canvas remains significantly faster than HTML and SVG animations, and its performance degradation is predictable and gradual. With DOM technologies (HTML/SVG), the results are lower, but the difference between CSS and JS approaches depends on the platform and activated features. HTML/JS and SVG/JS achieve higher FPS values than the CSS variants, but as the number of elements increases (especially above 1000), there is a gradual drop in framerate. With HTML/CSS and SVG/CSS, the FPS values are the lowest, and the drop occurs earlier, already at medium load, confirming the limitations of a large DOM and its repaint/rendering pipeline. The behaviour of iOS is particularly noteworthy, as in the measurements conducted, it maintains maximum FPS for an exceptionally long time, much longer than Windows, macOS, Linux, and especially Android. This effect is particularly evident in the variants without transformations (T). With random colours (C), iOS maintains the maximum frame rate even under higher loads compared to desktop operating systems. Only when blending (B) is enabled does iOS performance begin to decline noticeably, which aligns with Apple’s approach to off-screen compositing and alpha blending operations. Blending (B) proved to be the most critical factor for FPS across all technologies: when effects requiring additional compositing calculations are included, FPS drops most rapidly, especially in HTML and SVG animations. CSS animations are most sensitive to the combination of C, B, and T.

**Figure 8:**
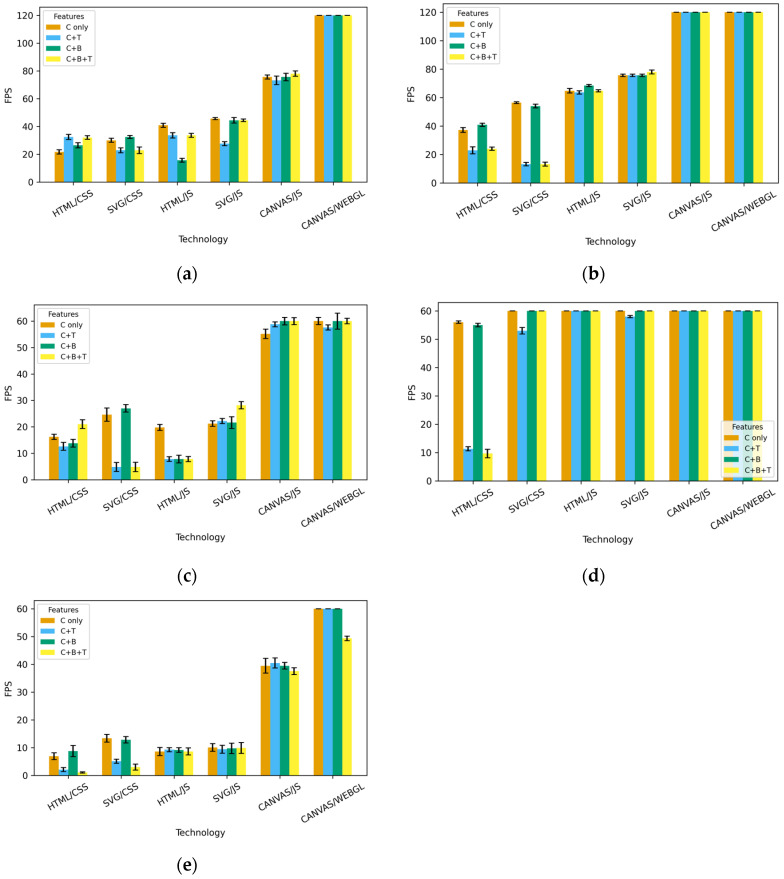
Comparison of FPS across web technologies, features, and all OSs on 5000 iterations: (**a**) Windows OS, (**b**) Mac, (**c**) Linux, (**d**) iOS, and (**e**) Android. Error bars indicate standard deviation across repeated runs (n = 10).

It is important to note the difference in the maximum number of frames per second with different monitors and their respective drivers (144/120/75/60), which results from the difference in screen capabilities in achieving certain refresh rates (Hz) on the devices used for the measurements. Monitors connected to the device can strongly influence the demand on CPUs or GPUs by requesting different frame loads for their resolution and different frame generation frequencies for their refresh-rate capabilities, but, as can be seen, those fixed differences were quickly annulled by the exponential growth of resource consumption demanded by animations in the test set. An overview of FPS by operating system, technology, and setting variations with 5000 iterations is shown in Figure 8 for Windows OS, macOS, Linux, iOS, and Android.

A table containing data from all research conducted across Windows, Linux, Mac, iOS, and Android operating systems using web technologies, including the number of iterations and additional settings, is available in the Supplementary section titled average_measured_data.

## 4. Discussion

The measurement results clearly show that the behaviour of web technologies under higher loads differs significantly between technologies, with deviations visible even before 5000 concurrencies. It is important to note that DOM-based technologies (HTML/CSS, SVG/CSS, HTML/JS, and SVG/JS) exhibit the most pronounced limitations as iterations increase, particularly when changing colours or including blending operations. These effects are consistent with the deliberately stress-oriented nature of the benchmark workload, which combines frequent repainting, compositing, and blending operations to reveal scaling limits and execution-path bottlenecks.

In several cases, with 5000 elements, HTML/CSS animations on Windows and Linux display slow loading, stretched animation frames, and delayed colour rendering. This effect is further intensified at 10,000 elements, where the animation becomes visually fragmented (with “ripped frames”), and DOM repaint cycles are delayed by several seconds.

SVG/CSS performs slightly better than HTML/CSS, as shown by the more stable FPS and the continuous display of animations, although with a longer initial load time. However, SVG also exhibits a clear decline in performance at higher loads, particularly in the blended variants.

With HTML/JS and SVG/JS animations, a specific behaviour was observed exclusively on Windows systems: at loads of 5000 and 10,000 elements, the browser repeatedly displayed a black screen for a few seconds before the animation reappeared. This phenomenon did not occur on macOS or Linux, although these systems showed higher GPU or CPU usage in certain segments. Despite the powerful hardware (i9 processor), this short-term “GPU reset” effect is typical when the render pipeline exceeds the allowed time threshold on the Windows platform. The reported OS-specific behaviours, including GPU reset events on Windows and prolonged load states on macOS and Linux, should be interpreted within the context of the experimental environment. Measurements were conducted in contemporary browsers using default OS and GPU driver configurations, without changes to TDR settings, power management policies, or driver-level parameters. Because of platform-specific release cycles, browser and driver versions were not identical across operating systems. Hardware configurations included both integrated and discrete GPUs, and thermal conditions were monitored but not explicitly measured. Therefore, the observed differences reflect real-world browser–OS–GPU interactions rather than isolated effects of individual system parameters.

Actionable guidance can be derived using measured intervals. With discrete object counts (10, 100, 500, 1000, 5000, 10,000), DOM-based techniques (HTML/CSS, SVG/CSS) remain stable at 100 objects, but consistent degradation is observed by 500 objects across several feature combinations, indicating a practical limit between 100 and 500 objects for sustained animation. These thresholds should be interpreted as upper limits under adverse animation conditions, rather than as representative performance for typical transform-only user interface animations, which are deliberately designed to avoid repaint-intensive operations.

Canvas-based techniques support higher object counts, while WebGL is preferred at the highest tested loads (5000–10,000 objects), where stability under heavy scenes is essential. As the exact breakpoint likely falls between adjacent sampling levels, identifying technology-specific breakpoints using finer-grained intermediate counts is proposed for future work.

Future work could use finer-grained object count increments between 100 and 500 (and between other adjacent levels) to identify technology-specific breakpoints with greater precision.

On mobile devices, where only FPS was measured, the differences are even more pronounced. On iOS, the iPhone (17 Pro) maintains 60 FPS under relatively high loads, but at 10,000 elements, the HTML/CSS animation freezes completely. This behaviour highlights the distinction between sustained perceptual smoothness and underlying system pressure, which is masked under stress-test conditions until abrupt failure occurs.

Loading takes much longer than on desktop platforms, and the animation display is delayed with occasional frame “bursting”. Similarly, HTML/JS and SVG/JS animations take a long time to load, and in some cases, the animation stops the machine from responding entirely, indicating a break in the rendering loop due to thermal or compositional limitations of WebKit. Unless explicitly stated otherwise, all reported thresholds and comparative conclusions are based on the primary measurement grid of up to 10,000 elements.

For further comparison, several extreme tests (100,000 and 1,000,000 elements) were conducted. These are not part of the standard set of measurements but serve to demonstrate the scalability ceiling. These extreme cases should be interpreted solely as stress-test boundaries rather than realistic application scenarios.

The results clearly show that DOM technologies cannot even initialise scenes of this size. HTML and SVG (both CSS and JS variants) fail to display animation stably on any operating system. On Windows, this often results in a brief black screen (indicating GPU reactivation), while macOS and Linux remain in a prolonged loading state without displaying anything. On iOS, the scene fails to initialise at 100,000 elements, causing the application to freeze. Canvas/JS is somewhat more resilient, but only for the first few seconds, after which the frame rate drops to 1–3 FPS. Only WebGL can initialise the scene at 100,000 objects, although with a very low frame rate (typically 1–5 FPS), while 1,000,000 elements exceed the practical rendering limits of all technologies.

Ultimately, the results clearly show that DOM-based animations have very limited tolerance for large numbers of elements in demanding scenarios such as those in this research, with serious compromises in visual continuity, loading time, and display stability already evident at 5000 elements. Canvas behaves more predictably but loses fluidity when trying to render more than 10,000 elements. WebGL is the only technology that maintains high performance and stability with several thousand elements, and it is the only one capable of initiating extreme tests, albeit with low FPS. Differences between operating systems show that Windows maintains performance most consistently at lower loads, iOS sustains maximum FPS the longest, although it freezes at very high loads, while macOS and Linux exhibit the earliest performance drops in DOM technologies, but without the GPU reset effects observed on the Windows platform. It is also notable that Linux and macOS isolate their processes during execution, so when a web browser process freezes, other computer processes, although mechanically slowed down for thermal regulation, continue running uninterrupted. This is not the case with Windows, where a browser freeze can cause several other unrelated programmes or services to stop, drastically degrading the user experience. Because the evaluated desktop platforms differ in hardware architecture, GPU class, and thermal design, the reported differences should be interpreted as observations on specific platform stacks (OS–browser–hardware combinations) rather than as isolated effects of the operating system.

Furthermore, when developing web applications, it is important to remember that HTML and SVG technologies are, in contrast to Canvas visualisations via JavaScript or WebGL, DOM-based. This makes their content programmatically searchable and manageable by accessibility readers. JavaScript animations and their triggers are fully customizable, while CSS-based approaches force development to conform to limitations of customizing only parameters for predefined functions and being able to trigger those animations only on timed intervals or state changes detectable by CSS selection mechanisms. Additionally, HTML is the only semantic-oriented markup technology native to web browsers, which often makes it the only viable technology for presenting informational content. Canvas rendering techniques, on the other hand, are pixel-based, and their objects cannot transmit computer-readable semantic content unless the application is built from the ground up to accommodate this shortcoming by communicating the necessary semantic content on demand to the required accessibility APIs. This limitation is not relevant when dealing with non-semantic content, such as stylistic decorations, and the performance benefits of Canvas rendering techniques make them advantageous in this context.

The results of this research confirm the findings of previous studies [2,12,13] on the limitations of DOM-based animation techniques, but significantly extend them by quantifying the thresholds for performance degradation. While CSS animations perform well at lower loads, serious display problems appear between 100 and 500 elements, and stability declines further with thousands of elements. Canvas shows more predictable behaviour at higher loads. WebGL is the only technology capable of initialising extremely complex scenes. The results also reveal significant differences between operating systems and devices, clearly confirming the need for cross-device and cross-OS benchmarking of web animation technologies.

The novelty of our approach lies in systematically combining common animation features, unlike prior studies, which typically evaluate isolated animation techniques. This method reflects realistic web scenarios and reveals interaction effects—such as the disproportionate impact of blending under high object counts—that are not apparent in simpler benchmark designs.

By presenting the benchmark as a stress-oriented workload, the results complement existing representative UI studies by providing upper-bound performance characteristics and identifying where rendering paths no longer remain perceptually stable under worst-case demands.

## 5. Conclusions

This study shows that web animation performance depends heavily on the rendering model, the underlying platform, and the level of visual complexity. DOM-based technologies, while easy to implement and suitable for small scenes, deteriorate rapidly under higher loads. Canvas provides more predictable behaviour and maintains acceptable fluidity with up to several thousand elements, whereas WebGL is the only technology capable of supporting large-scale animations and handling extreme scenarios. Platform differences are significant: Windows exhibits the highest stability at moderate loads; macOS and Linux degrade sooner but more gracefully; and iOS maintains smooth playback for longer but fails abruptly at very high loads. It is important to emphasise that these conclusions are based on a deliberately stress-oriented benchmark workload that combines frequent repainting, compositing, and blending operations. The reported thresholds, therefore, represent upper-bound performance under adverse rendering conditions, rather than average user interface animation performance. For mobile platforms, performance evaluation is limited to FPS measurements due to the lack of reliable browser-level CPU and GPU instrumentation. Cross-platform comparisons should be interpreted with this limitation in mind. Overall, the results offer clear guidance for choosing rendering technologies based on the intended scale and performance requirements.

Future research should broaden the scope of this work to achieve a more comprehensive understanding of performance characteristics in real-world environments. First, mobile platforms require more thorough investigation. The current measurements were constrained by the absence of browser-level diagnostic tools capable of capturing low-level metrics such as CPU and GPU activity, thermal throttling, memory allocation, and frame scheduling on iOS and Android. Although numerous performance-profiling tools are available for native games, equivalent instrumentation for mobile browsers remains underdeveloped. Developing or adapting tools to capture these metrics in an open browser context would significantly enhance future analyses.

Based on the measured CPU, GPU, and FPS behaviour across rendering technologies and operating systems, the results of this study can be interpreted as practical recommendations for developers of web animations, browser-based games, and interactive educational content. These recommendations should be applied with the understanding that many production UI animations deliberately use transform-only motion and opacity changes to minimise repaint costs, and may therefore demonstrate more favourable performance than the stress-test scenarios evaluated here. However, as described in the Discussion, all web-native animation technologies have both advantages and disadvantages. Neither fully supersedes the other in all respects. DOM-based technologies provide content searchability and other accessibility benefits, which Canvas-based ones lack. Among these, SVG lacks standardised semantic markup definitions, while HTML allows only block-shaped objects when composing layouts. JavaScript-based techniques enable detailed interaction design, while CSS-based approaches offer only an interface for customising parameters of predefined layout and animation functions, and can be triggered only by a limited set of cursor interactions or timed events. All web animation technologies degrade the user experience in specific ways when their computational requirements exceed available resources. All these considerations need to be taken into account when choosing the optimal web animation approach for a specific use case. Understanding the performance differences in animation rendering between different web-native animation technologies, as discussed in this paper, contributes to the complexity and quality of this decision-making process. Accordingly, the guidance provided by this study is intended to inform technology selection based on known workload characteristics, rather than to prescribe universal best practices.

CSS- and SVG-based approaches show low GPU utilisation but increased CPU instability at higher object counts, making them suitable for low-complexity visualisations and instructional graphics rather than sustained interactive workloads. In contrast, Canvas- and WebGL-based implementations demonstrate greater performance stability under high iteration counts and continuous interaction, which is particularly relevant for real-time gameplay mechanics and interactive learning environments.

Furthermore, OS-specific performance characteristics indicate that cross-platform development should consider system-level recovery behaviour and resource management. Adaptive strategies such as dynamic scene complexity, feature scaling, or runtime degradation can help maintain responsiveness across desktop and mobile platforms in both gaming and educational contexts.

Battery consumption is another important aspect that should be quantified. High-load animations affect energy usage differently across rendering technologies, and understanding these differences is increasingly relevant given the growing reliance on mobile web applications. Similarly, thermal behaviour and device heat buildup directly impact throttling events and user comfort; systematic measurements of device temperature under controlled animation loads would help identify safe operating thresholds.

Future work should also consider defining safety guidelines to prevent device overload, particularly when rendering thousands of parallel objects. Such guidance could include recommended limits for DOM elements, warnings for Canvas and WebGL scenarios approaching thermal or computational limits, and platform-specific recommendations to avoid GPU resets (such as TDR events observed on Windows).

Additionally, research could explore how to preserve a positive user experience in highly complex animation environments. This includes strategies for adaptive degradation (such as dynamically reducing visual detail), multi-layer rendering pipelines, hybrid DOM–Canvas/WebGL approaches, and the use of Web Workers or Offscreen Canvas for parallel computation. Large-scale animations involving 2D/3D hybrid scenes, semantic object grouping, or AI-assisted optimization methods may also offer new opportunities to maintain both performance and visual quality.

## Figures and Tables

**Table 1:** Showing the most important specifications of the devices on which measurements were performed.

Device			CPU		GPU	RAM	Monitor Hz
ABRV.	Device Name	OS	Device	Cores	Device		
Windows	ASUS Zenbook UX8402ZE	Windows11 25H2	12th Gen Intel i9-12900H	14	Nvidia GeForce RTX 3050Ti	32	120
Linux	Lenovo ThinkPad T14	Pop!_OS 24.04 LTS	11th Gen Intel i7-1165G7	4	Intel TigerLake-LP GT2	48	60
Mac	Mac Mini (16,10)	MacOS Tahoe	Apple M4	10	Apple M4	16	120
iOS	iPhone 17 Pro	iOS 26.1	A19 Pro	6	A19 Pro	12	60
Android	Galaxy A52s 5G	Android 14	Snapdragon 778G 5G	8	Adreno 642L	8	60

**Table 2:** Showing browser versions across operating systems.

Operating System	Chrome Version
Windows	143.0.7499.170
Linux	143.0.7499.169-1
Mac	143.0.7499.169
iOS	143.0.7499.151
Android	143.0.7499.146

## Data Availability

The original contributions presented in this study are included in the article/Supplementary Material. Further inquiries can be directed to the corresponding author.

## Supplementary Materials

The following supporting information can be downloaded at: https://www.mdpi.com/article/10.3390/jimaging12010045/s1, Excel file *average_measured_data*, text file links containing links to the testing app and documentation.
